# Supplementary material for: Effectiveness of Integrative Korean Medicine Treatment in Patients with Traffic-Accident-Induced Acute Low Back Pain and Mild Adult Scoliosis
Source: Healthcare (Basel). 2023 Jun 13;11(12):1735. doi: 10.3390/healthcare11121735 (PMC10298022; doi:10.3390/healthcare11121735)
Supplement: Supplementary file 1 [file healthcare-11-01735-s001.zip › healthcare-2438628-supplementary.pdf]

Supplementary Table S1. Basic participant characteristics in the total group

	Total		
		Cobb's Angle ≤ 20	Cobb's Angle > 20
	N=674	N=597	N=77
**Age (years)**			
Mean ± SD	38.05 ± 14.01	38.22 ± 13.99	36.75 ± 14.20
Less than 30	263 (39.0)	227 (38.1)	36 (46.8)
30-50	230 (34.2)	207 (34.6)	23 (29.9)
More than 50	181 (26.8)	163 (27.3)	18 (23.4)
**Sex**			
Male	226 (33.5)	210 (35.2)	16 (20.8)
Female	448 (66.5)	387 (64.8)	61 (79.2)
**Cobb's Angle, degrees**			
10~20	597 (88.6)	597 (100.0)	-
21~35	69 (10.2)	-	69 (89.6)
36~40	3 (0.4)	-	3 (3.9)
41~50	1 (0.1)	-	1 (1.3)
51~55	1 (0.1)	-	1 (1.3)
More than 56	3 (0.4)	-	3 (3.9)
**Height (Total N=670)**			
Mean ± SD	165.68 ± 8.44	166.04 ± 8.47	162.87 ± 7.68
**Weight (Total N=670)**			
Mean ± SD	63.11 ± 14.16	63.80 ± 14.49	57.74 ± 9.84
**BMI (Total N=670)**			
Less than 25	489 (73.0)	427 (72.0)	62 (80.5)
More than 25	181 (27.0)	166 (28.0)	15 (19.5)
**Comorbidity**			
Hypertension	65 (9.6)	58 (9.7)	7 (9.1)

Diabetes Mellitus	30 (4.5)	27 (4.5)	3 (3.9)
Depressive disorder	29 (4.3)	26 (4.4)	3 (3.9)
Cardiovascular Disease	70 (10.4)	62 (10.4)	8 (10.4)
Respiratory disease	43 (6.4)	39 (6.5)	4 (5.2)
Gastrointestinal disease	82 (12.2)	74 (12.4)	8 (10.4)
Etc	253 (37.5)	219 (36.7)	34 (44.2)
**Smoking**			
Yes	151 (22.4)	138 (23.1)	13 (16.9)
No	523 (77.6)	459 (76.9)	64 (83.1)
**Alcohol use**			
Yes	195 (28.9)	173 (29.0)	22 (28.6)
No	479 (71.1)	424 (71.0)	55 (71.4)
**Outcomes at admission**			
Admission_LBP for NRS*	4.90 ± 0.81	4.87 ± 0.77	5.08 ± 1.02
Admission_EQ-5D-5L*	0.68 ± 0.14	0.68 ± 0.14	0.68 ± 0.14
Admission_ODI*	35.90 ± 13.95	35.80 ± 13.89	36.65 ± 14.46
Admission ROM flexion* (Total N=671)	89.04 ± 5.47	89.14 ± 5.21	88.22 ± 7.20
Admission ROM extension* (Total N=671)	19.61 ± 2.16	19.63 ± 2.14	19.41 ± 2.30

Supplementary Table S2. Treatment details during the hospital stay

Variable	Total (n=674)	Follow-up (n=101)
**Length of hospital stay (mean SD)**	**9.24 ± 3.92**	**9.48 ± 4.26**
**Acupuncture**		
Treatment provided	674 (100.0)	101 (100.0)
Number of sessions	15.68 ± 7.75	16.25 ± 8.35
**Electropuncture**		
Treatment provided	674 (100.0)	101 (100.0)
Number of sessions	15.57 ± 7.73	16.20 ± 8.36
**Pharmacopuncture**		
Treatment provided	664 (98.5)	99 (98.0)
Number of sessions	6.52 ± 2.48	6.65 ± 2.87
**Herbal medicine**		
Treatment provided	670 (99.4)	100 (99.0)
Number of sessions	17.36 ± 8.13	17.33 ± 9.27
**Chuna**		
Treatment provided	613 (90.9)	90 (89.1)
Number of sessions	7.16 ± 4.43	7.44 ± 4.82
**Cupping**		
Treatment provided	674 (100.0)	101 (100.0)
Number of sessions	15.65 ± 7.74	16.23 ± 8.35
**Moxibustion**		
Treatment provided	546 (81.0)	87 (86.1)
Number of sessions	6.36 ± 5.52	7.30 ± 6.35

Supplementary Table S3. Changes in outcomes from baseline for the total group

	**Admission (baseline)**	**Discharge**	**Follow-up**
NRS_LBP			
Value	4.90 (4.84 to 4.96)	3.52 (3.43 to 3.61)	3.03 (2.81 to 3.26)
Diff	—	1.38 (1.29 to 1.47)	1.87 (1.64 to 2.09)
p-value		<0.001	<0.001
ODI			
Value	35.90 (34.85 to 36.95)	23.63 (22.77 to 24.49)	14.23 (12.17 to 16.30)
Diff	—	12.27 (11.41 to 13.13)	21.67 (19.60 to 23.73)
p-value		<0.001	<0.001
EQ-5D-5L			
Value	0.68 (0.67 to 0.69)	0.78 (0.77 to 0.78)	0.89 (0.87 to 0.91)
Diff	—	-0.09 (-0.10 to -0.09)	-0.21 (-0.22 to -0.19)
p-value		<0.001	<0.001

Values are presented as least square means and 95% confidence intervals. Differences from baseline and p-values were calculated using a linear mixed model adjusted for baseline outcome, sex, and age.

NRS, numeric rating scale; ODI, Oswestry Disability Index; EQ-5D-5L, 5-level EuroQol 5-dimension; Diff, the difference from baseline.

Supplementary Table S4. Differences in outcomes according to Cobb's angle in the total group

	Admission (baseline)	Discharge	Follow-up
NRS_LBP			
Cobb's ≤20°	4.90 (4.84 to 4.96)	3.38 (3.12 to 3.64)	3.12 (2.47 to 3.77)
Cobb's >20°		3.54 (3.44 to 3.63)	3.02 (2.78 to 3.26)
Difference*	—	-0.16 (-0.43 to 0.11)	0.10 (-0.59 to 0.79)
P value		0.255	0.771
ODI			
Cobb's ≤ 20°	35.90 (34.85 to 36.95)	23.98 (21.47 to 26.50)	13.27 (7.31 to 19.23)
Cobb's >20°		23.59 (22.68 to 24.49)	14.37 (12.17 to 16.57)
Difference*	—	0.39 (-2.23 to 3.02)	-1.10 (-7.44 to 5.25)
P value		0.768	0.734
EQ-5D-5L			
Cobb's ≤ 20°	0.68 (0.67 to 0.69)	0.79 (0.77 to 0.80)	0.88 (0.84 to 0.93)
Cobb's >20°		0.78 (0.77 to 0.78)	0.89 (0.87 to 0.91)
Difference*	—	0.01 (-0.01 to 0.03)	-0.01 (-0.06 to 0.04)
P value		0.303	0.697

The mean baseline outcomes were calculated by pooling the baseline values between the two groups. The outcomes at discharge and follow-up in the two groups are presented as least square means, and 95% CI is calculated using a linear mixed model. The difference refers to the difference in the change from baseline between the two groups. Differences and p-values were calculated using a linear mixed model adjusted for baseline outcome, sex, and age.

NRS, numeric rating scale; ODI, Oswestry disability index; EQ-5D-5L, 5-level EuroQol 5-dimension

Supplementary Table S5. Factors associated with improvements in NRS scores for back pain and ODI in the total group

	NRS	ODI
	Discharge (n = 670)	Discharge (n = 670)
Number of patients who achieved the MCID	232	294
The baseline value of each variable	2.14(1.66-2.74)	1.26(1.04-1.54)
Age (ref. < 30 years)		
30–49 years	0.65(0.43-0.98)	0.89(0.62-1.30)
≥ 50 years	1.11(0.73-1.69)	0.81(0.55-1.20)
Sex (ref. male)		
Female	1.24(0.82-1.88)	0.72(0.49-1.05)
BMI (ref. BMI < 25)		
BMI ≥25	1.71(1.15-2.54)	0.89(0.62-1.29)
Smoking (ref. No)		
Yes	0.66(0.41-1.05)	0.75(0.49-1.16)
Drinking (ref. No)		
Yes	1.27(0.85-1.90)	0.71(0.49-1.03)
Cobb's angle (ref. ≤ 20)		
>20	1.56(0.93-2.64)	0.71(0.43-1.18)
**AUC (95% CI)**	0.67(0.63-0.71)	0.55(0.51-0.60)
